# The complete chloroplast genome and phylogenetic analysis of *Citrus clementina* (Rutaceae)

**DOI:** 10.1080/23802359.2021.1972860

**Published:** 2021-09-13

**Authors:** Changlan Sun, Haifeng Lin

**Affiliations:** School of Information Science and Technology, Nanjing Forestry University, Nanjing, China

**Keywords:** Rutaceae; *Citrus clementina*, chloroplast genome, phylogenetic analysis

## Abstract

Citrus, which is widely cultivated in tropical and subtropical climates, is one of the most important crops in the world. Here, we assembled and annotated the complete chloroplast genome of *Citrus clementina* (*C. clementina*) using Illumina sequencing data. This chloroplast genome exhibited a typical quadripartite structure of 154,042 bp in length, and the overall GC content was 38.42%. A total of 134 genes (90 protein-coding genes, 36 tRNAs, and eight rRNAs) were predicted in this chloroplast genome. Phylogenetic analysis strongly supported that *C. clementina* was evolutionarily close to *Citrus sunki*.

A clementine (*Citrus clementina*) is a citrus fruit hybrid between a mandarin orange and a sweet orange (Wu et al. 2014; Shimizu et al. 2016). Clementines have a looser skin that makes them easier to peel than oranges, though they are smaller than oranges. Additionally, they are sweeter and have more vitamin C than other mandarins and oranges. The scaffold-level genome (size: 301.4 Mb, scaffolds: 1398, N50: 110 kb) of *C. clementina* has been assembled using a Sanger whole genome shotgun approach in 2013 (Wu et al. 2014), but the chloroplast genome has not been published so far. The sequence and structure of different chloroplast genomes are very conserved, which will provide important genomic resources for further species identification and evolutionary study (Wang et al. 2018). In this study, the complete chloroplast genome of *Citrus clementina* was assembled and characterized, which will facilitate the taxonomic and evolutionary studies of the genus Citrus.

Leaf sample of *C. clementina* was collected from Citrus Research Institute of Chinese Academy of Agricultural Sciences (CAAS), Chongqing, China (29°45′36.2″N, 106°22′40.5″E), and the voucher specimen was deposited at the Herbarium of Nanjing Forestry University under the voucher number 20171221_CC01 (Haifeng Lin, haifeng.lin@njfu.edu.cn). Genomic DNA was extracted from leaves using hexadecyl trimethyl ammonium bromide (CTAB) DNA extraction protocol (Bubner et al. 2004) and then sequenced using Illumina HiSeq 4000 platform. The DNA quantity and quality were assessed by NanoDrop spectrophotometer and 1% agarose gel electrophoresis, respectively. The raw sequencing data were first filtered and trimmed by fastp program (Chen et al. 2018), and then fed into NOVOPlasty v4.2.1 (Dierckxsens et al. 2017) for assembly using the reference chloroplast genome sequence of *Citrus sinensis* (GenBank accession no.: NC_008334.1). The assembled genome was then annotated using PGA (Qu et al. 2019) and corrected manually using Macvector v18.1 (Bi et al. 2016), and finally submitted to NCBI GenBank under the accession number MW207298.

The chloroplast genome of *C. clementina* displayed a typical quadripartite structure of most angiosperm chloroplast genomes, composing of one large single-copy region (LSC: 87,879 bp), one small single-copy region (SSC: 18,779 bp), and a pair of inverted repeats (IRs: 27,022 bp). The overall GC content of the chloroplast genome was 38.42%, higher than that of LSC (36.79%) and SSC (33.12%), but lower than IRs (42.92%). The chloroplast genome of *C. clementina* encoded a total of 134 functional genes, including 90 protein-coding genes, 36 tRNA genes, and eight rRNA genes. Among these, 20 genes contained one intron (12 protein-coding genes and eight tRNA genes), and two genes contain two introns (*ycf3* and *clpP*). To determine the phylogenetic relationships of *C. clementina*, we downloaded 16 other chloroplast sequences of genus Citrus and two species in Sapindales from the NCBI GenBank database. *Anacardium occidentale* and *Dimocarpus longan* were chosen as outgroup. A maximum-likelihood (ML) tree was constructed based on 76 conserved protein-coding genes of 19 cp genomes by RAxML (Stamatakis 2014) in CIPRES Science Gateway (Miller et al. 2010) with 1000 bootstrap replicates. The phylogenetic tree strongly supported that *C. clementina* was closely related to *Citrus sunki* (Figure 1). The complete chloroplast genome of *C. clementina* will provide important genetic and genomic resources to facilitate the molecular taxonomy and breeding of the genus Citrus.

**Figure 1.**
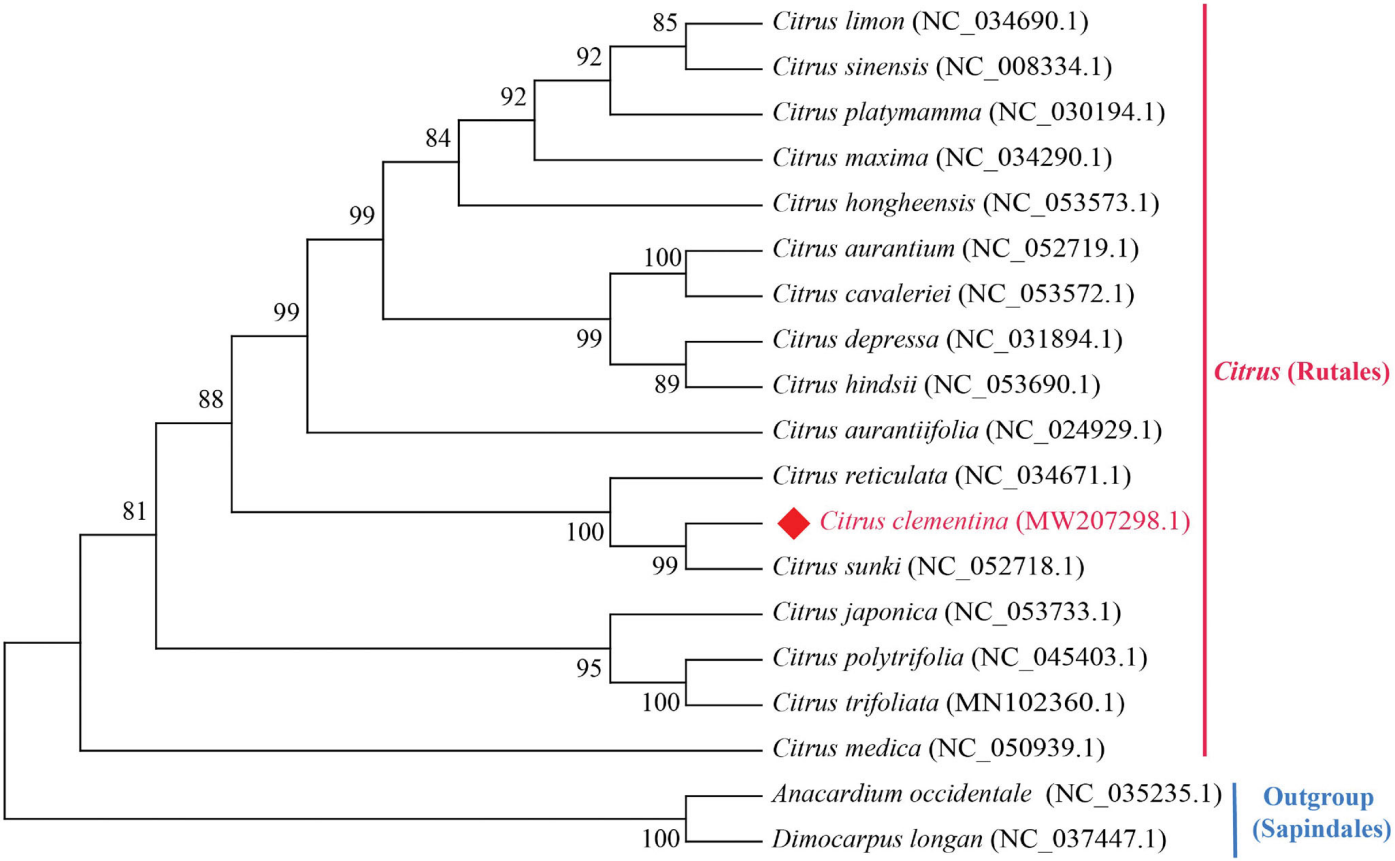
The maximum-likelihood (ML) phylogenetic tree of 19 plant chloroplast genomes is based on 76 conserved protein-coding genes. Numbers in the nodes are bootstrap values from 1000 replicates. The GenBank accession numbers for tree reconstruction are listed right to their scientific names.

## Data Availability

The genome sequence data that support the findings of this study are openly available in NCBI GenBank at https://www.ncbi.nlm.nih.gov/nuccore/MW2072968. The associated BioProject, SRA, and Biosample numbers are PRJNA422948, SRR6415870, and SAMN08222600, respectively.
